# Supplementary material for: Synthesis, Optical Properties, and Fluorescence Cell Imaging of Novel Mixed Fluorinated Subphthalocyanines
Source: Molecules. 2023 Jan 11;28(2):725. doi: 10.3390/molecules28020725 (PMC9866271; doi:10.3390/molecules28020725)
Supplement: Supplementary file 1 [file molecules-28-00725-s001.zip › molecules-2108791-supplementary.pdf]

Synthesis, Optical Properties and Fluorescence Cell Imaging of Novel Mixed Fluorinated Subphthalocyanines

Shutong Zhou[1], Xiaojuan Lv[2], Minghui Li[1], Zijian Gao[1], Shengnan Tu[1], Shanshan Qiao[1], Mengjia Mo[1], Xu Tang[3], Yemei, Wang[1]*, Shasha Sun[1]*

[1]School of Environmental and Chemical Engineering, Jiangsu University of Science and Technology, Zhenjiang 212013, China
[2]School of Chemistry and Chemical Engineering, Jiangsu University, Zhenjiang 212013, China
[3]Institute for Advanced Materials, School of Materials Science and Engineering, Jiangsu University, Zhenjiang 212013, China
*Correspondence: wangym@just.edu.cn, Tel.: +86-15365996930; sunshasha@just.edu.cn;

Supplementary Materials

Table of Contents

i. Experimental Section
ii. HRMS Spectra
iii. NMR Spectra
iv. IR Spectra
v. Crystallographic Data
vi. TD-DFT Calculation Results
vii. References

i. Experimental Section

General information: Unless noted, all reagents or solvents were obtained from commercial suppliers and used without further purification. All air sensitive experiments were performed in N_2 atmosphere through Schlenk technology. ^{1}H, ^{13}C, ^{19}F and ^{11}B NMR spectra were recorded on a JNM-ECZ400S/L1 spectrometer (operating as 400 MHz for ^{1}H, 100 MHz for ^{13}C, 376 MHz for ^{19}F and 128 MHz for ^{11}B) using the residual solvent as the internal reference for ^{1}H (δ = 7.26 ppm in $CDCl_3$), ^{13}C (δ = 76.8 ppm in $CDCl_3$), and CF_3COOH as the external reference for ^{19}F (δ = −76.5 ppm). ^{1}H and ^{19}F NMR spectra were acquired with Single Plus Experiment (SPE) with a pluse length of 3.06 μs and a recycle delay of 5 s. ^{13}C NMR spectra were acquired with Single Plus Experiment (SPE) with a pluse length of 3.30 μs and a recycle delay of 2 s. ^{11}B NMR spectra were acquired with Single Plus Experiment (SPE) with a pluse length of 3.33 μs and a recycle delay of 2 s. Mass spectra were conducted at Thermo Scientific Q Exactive Focus LC-MS/MS（APCI）. A Hitachi F-4600 fluorescence spectrophotometer was used to measure fluorescence spectrum. An Edinburgh FLS-980 spectrometer was used to determine fluorescence quantum yield. The Fourier transform infrared (FT-IR) spectra of the samples were recorded on a Thermo Scientific Nicolet iS50 spectrometer in the spectral range of 4000–400 cm^{-1} with 16 scans and a resolution of 8 cm^{-1}. Melting point of samples were recorded on a melting point tester of Tianfen-RY-51. Preparative separations were performed using silica gel column chromatography (Shanghai test Silica Gel 60N, spherical, neutral, 300-400 mesh). Thin-layer chromatography (TLC) was performed with aluminum sheet silica gel 60 F_{254} (Merck).

X–ray Analysis: Single crystal of **6** was obtained by the slow diffusion of n-hexane into a CH_2Cl_2 solution of them. X-ray crystallographic data for **6** (CCDC: 2220751) was recorded at 296 K on a Bruck APEX DUO diffractometer using graphite-monochromate Mo–Kα radiation from the corresponding set of confocal optics. The structures were solved by a direct method (SHELXT)[s1] and refined using a full-matrix least squares technique (SHELXL) [s2, s3].

Cyclic voltammetry: The cyclic voltammetry was conducted in a solution of 0.1 M TBAP in dry-CH_2Cl_2 with a scan rate of 0.1 Vs^{-1} in an argon-filled cell. A glassy carbon electrode and a platinum wire were used as a working and a counter electrode, respectively. A saturated Calomel electrode (SCE) was used as reference electrodes.

Computational methods: Gaussian 9 software packages were used to carry out density functional theory (DFT) and time-dependent DFT (TDDFT) calculations using a B3LYP functional with a 6-31G(d) basis sets.

Cell culture: For cell culture, cells were seeded in cell culture plates using Dulbecco's modified Eagle's medium (DMEM) with 10% fetal bovine serum (FBS) and incubated at 37% under a humidified atmosphere with 5% CO_2 for 24 h to reach 80% confluence.

ii. HRMS Spectra

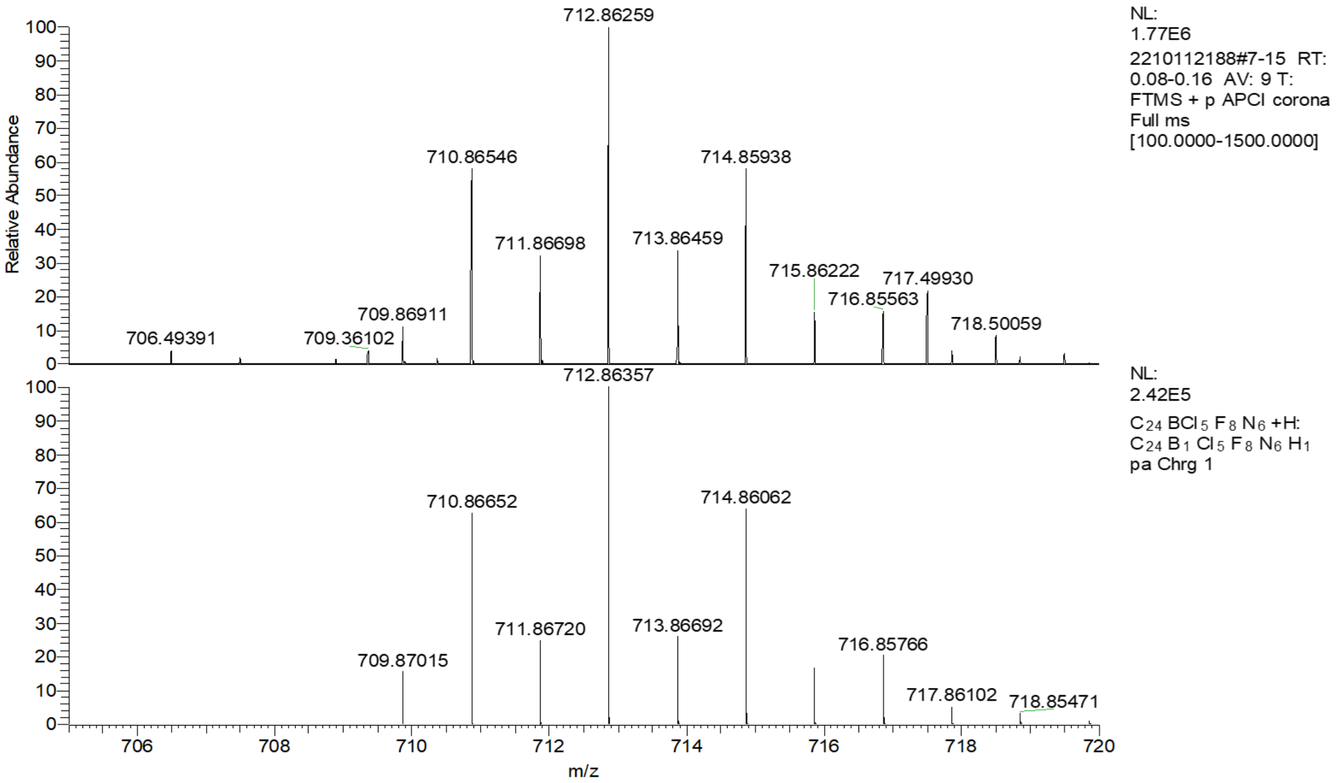

Figure S1. HR-APCI-MS spectrum of **5**.

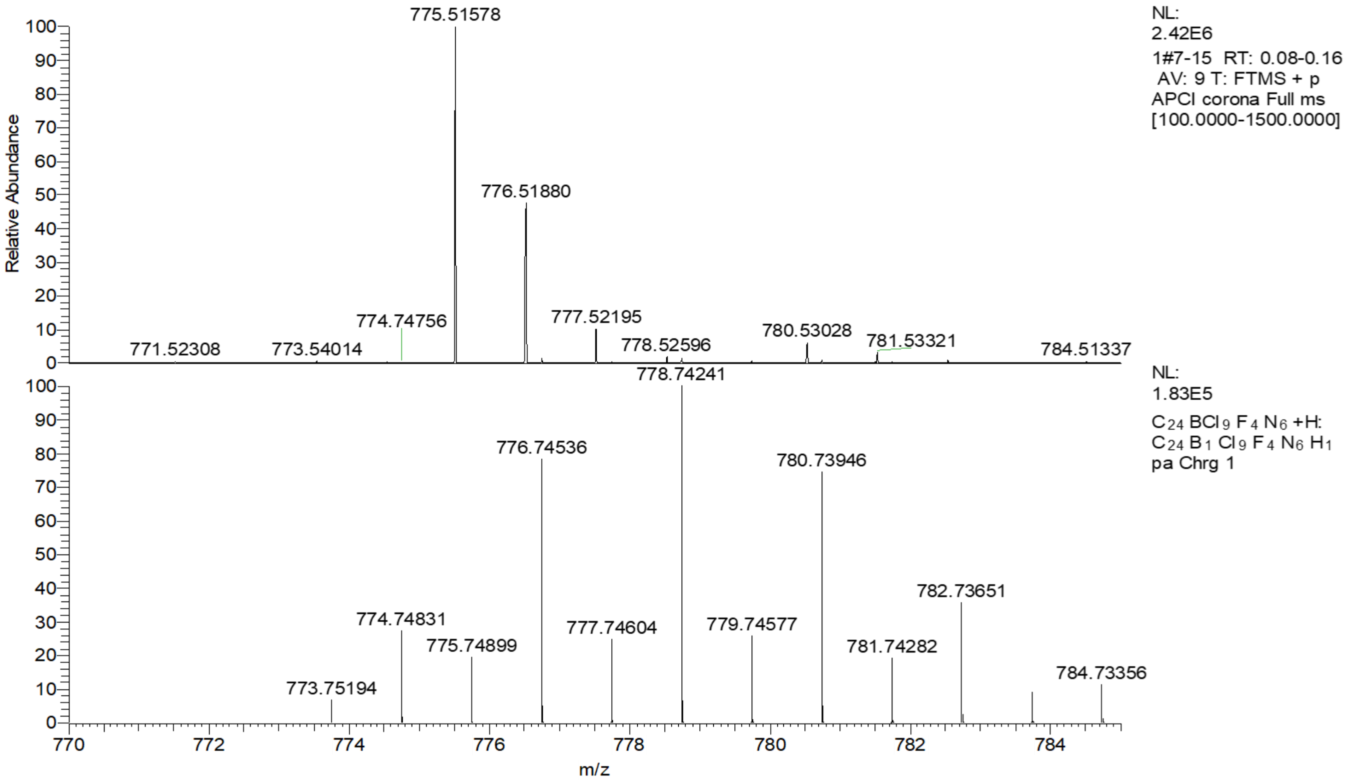

Figure S2. HR-APCI-MS spectrum of **6**.

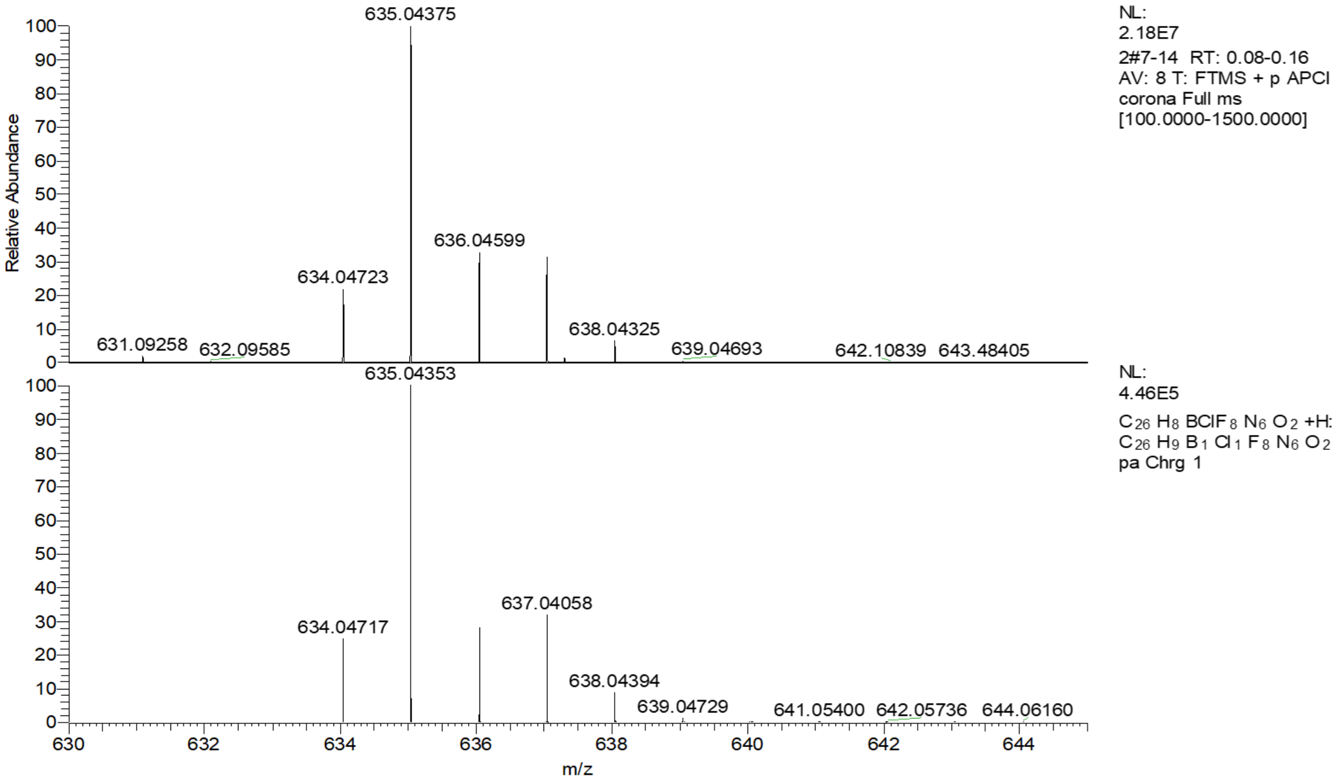

Figure S3. HR-APCI-MS spectrum of **7**.

iii. NMR Spectra

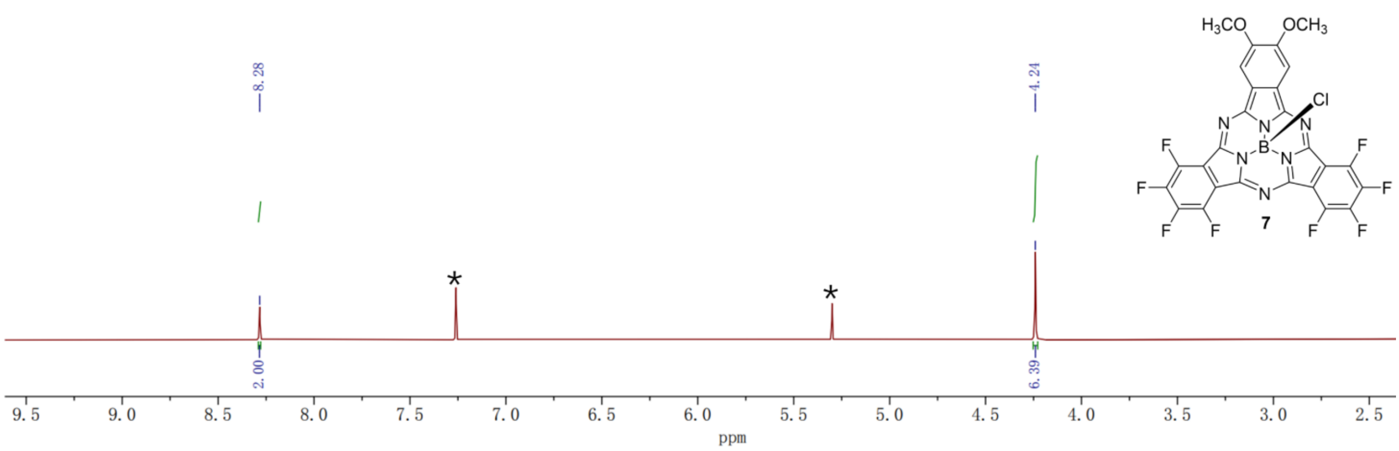

Figure S4. ^{1}H NMR spectrum of **7** in $CDCl_3$ (* residual solvent).

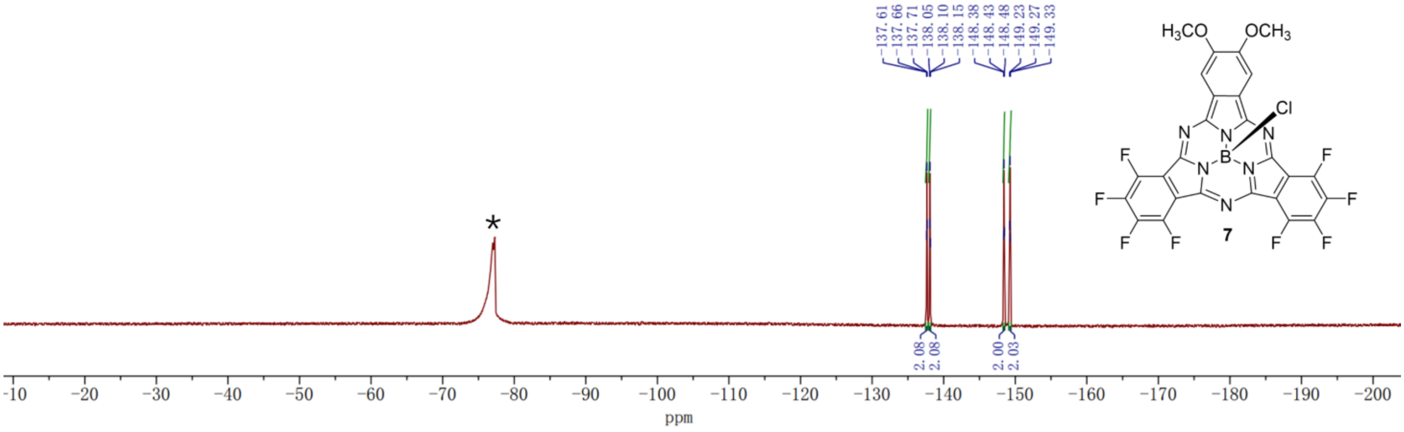

Figure S5. ^{19}F NMR spectrum of **7** in $CDCl_3$ (* CF_3COOH as reference).

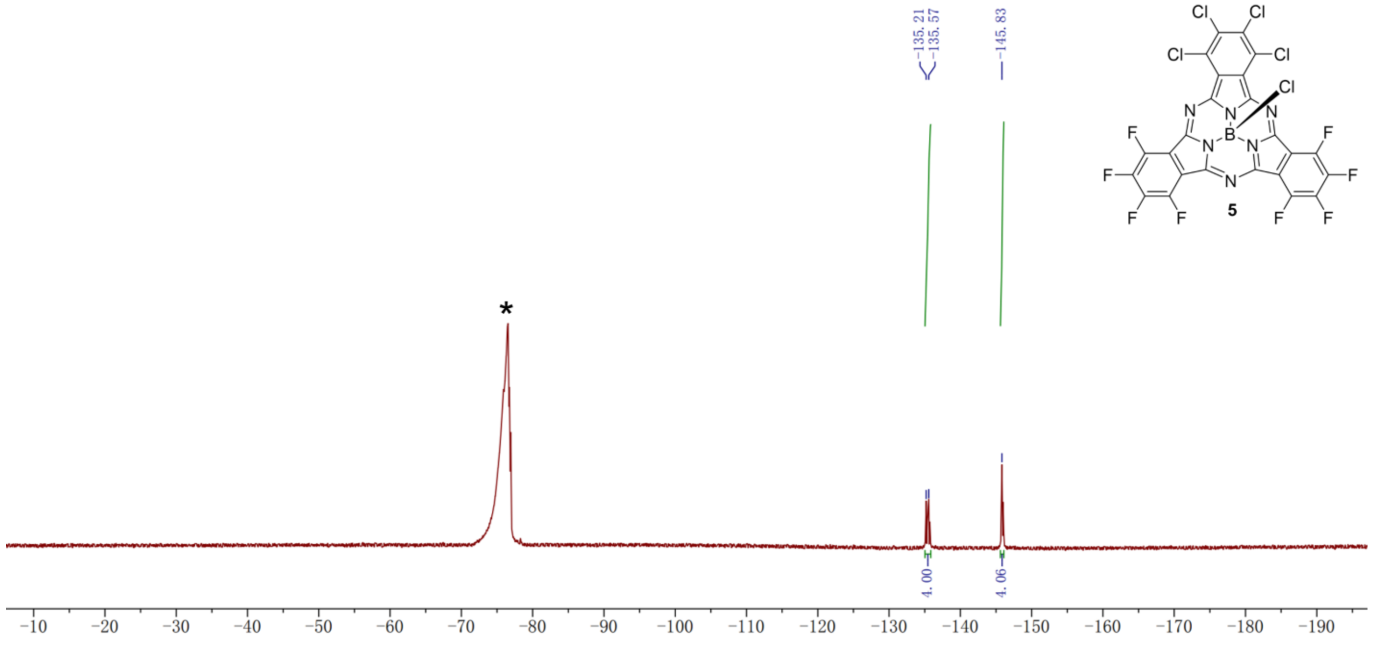

Figure S6. ^{19}F NMR spectrum of **5** in $CDCl_3$ (* CF_3COOH as reference).

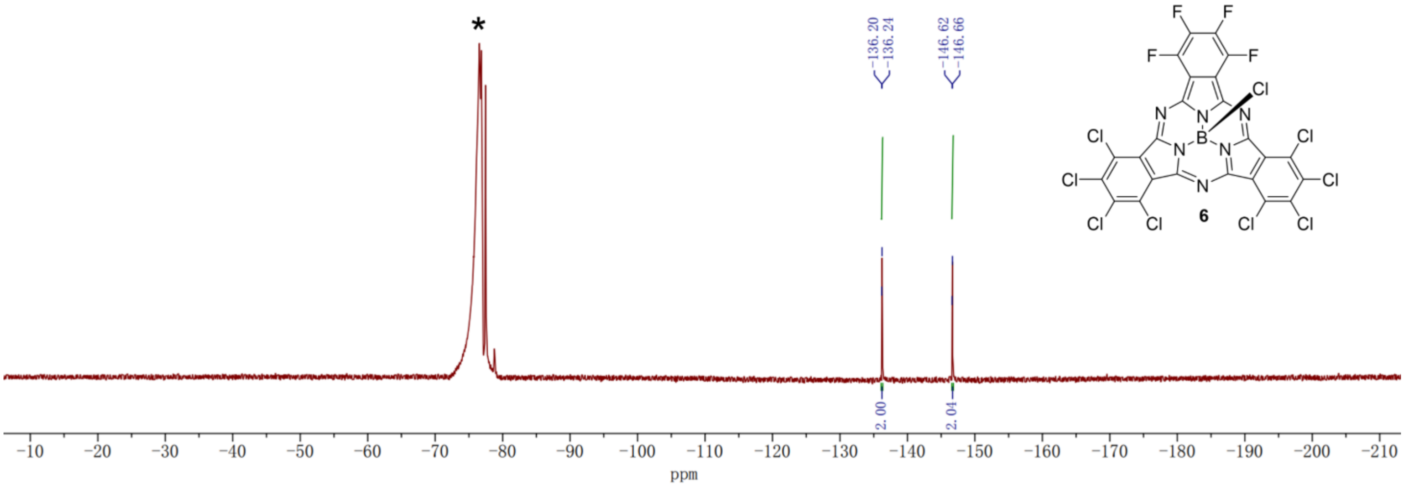

Figure S7. ^{19}F NMR spectrum of **6** in $CDCl_3$ (* CF_3COOH as reference).

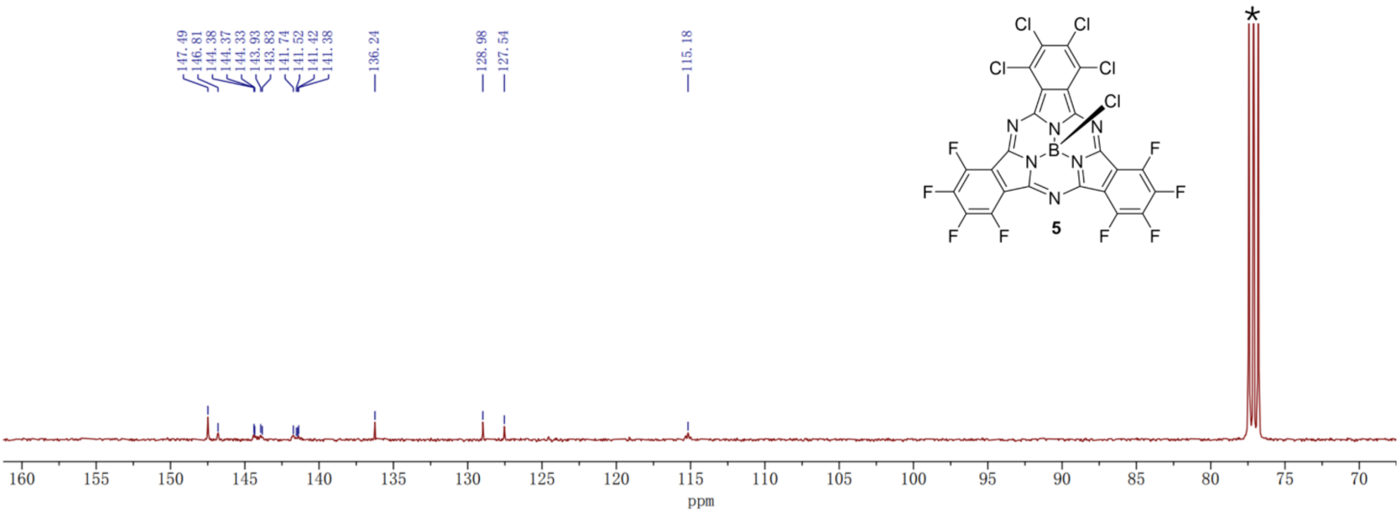

Figure S8. ^{13}C NMR spectrum of **5** in $CDCl_3$ (* residual solvent).

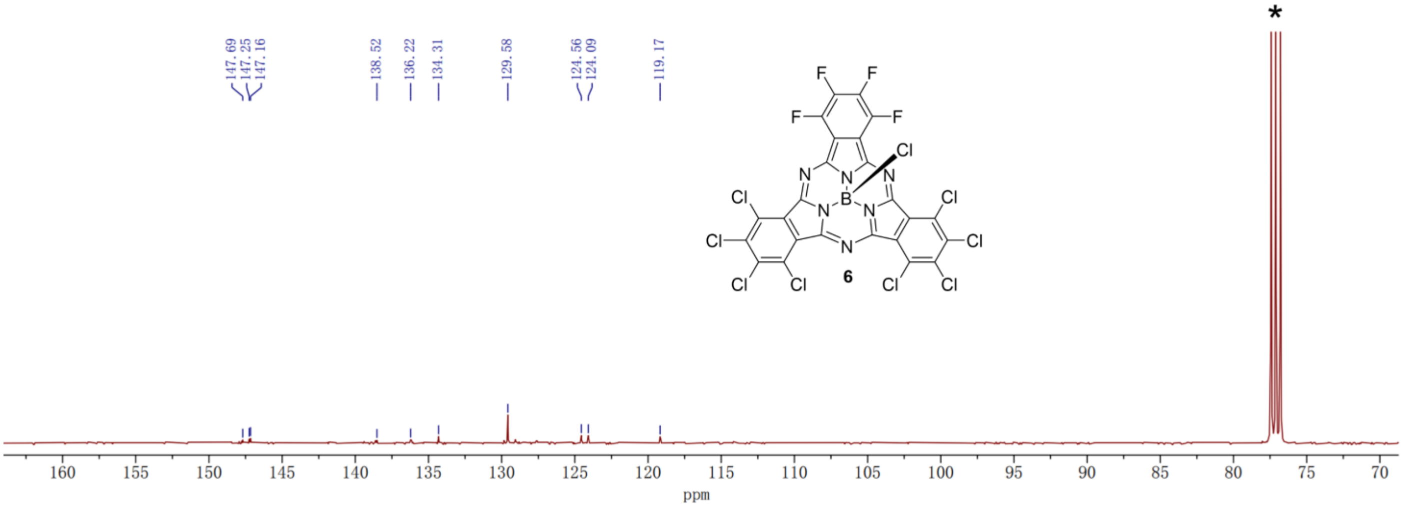

Figure S9. ^{13}C NMR spectrum of **6** in $CDCl_3$ (* residual solvent).

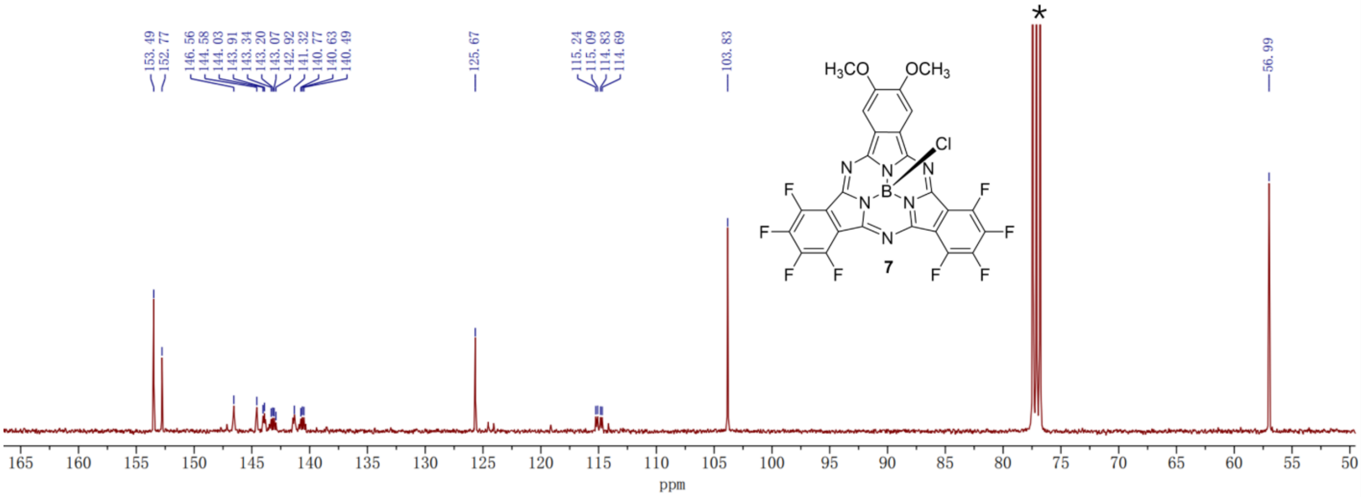

Figure S10. ^{13}C NMR spectrum of **7** in $CDCl_3$ (* residual solvent).

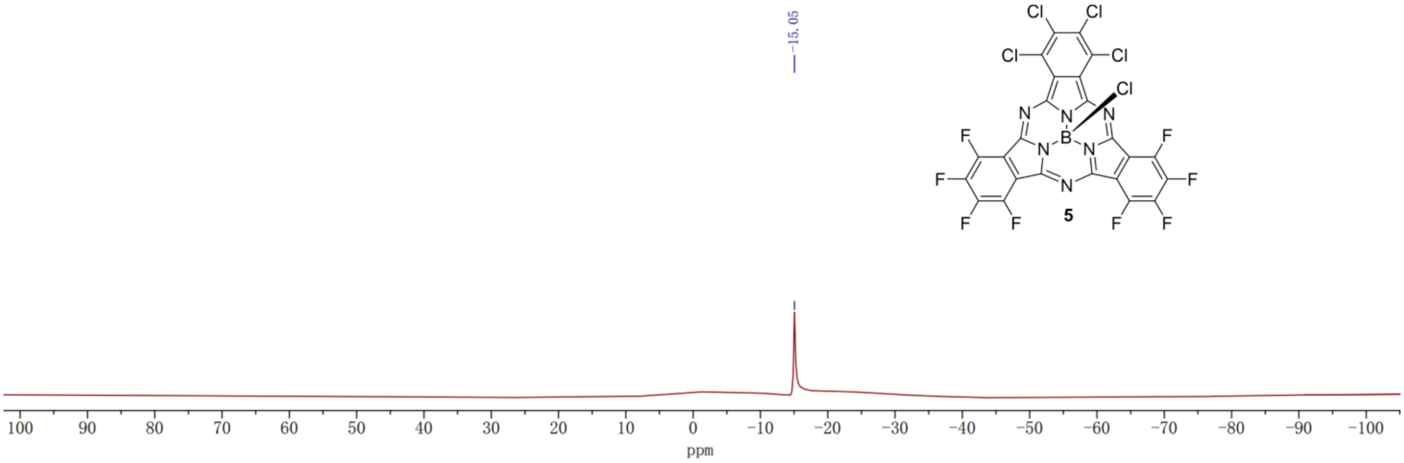

Figure S11. ^{11}B NMR spectrum of **5** in $CDCl_3$ (* residual solvent).

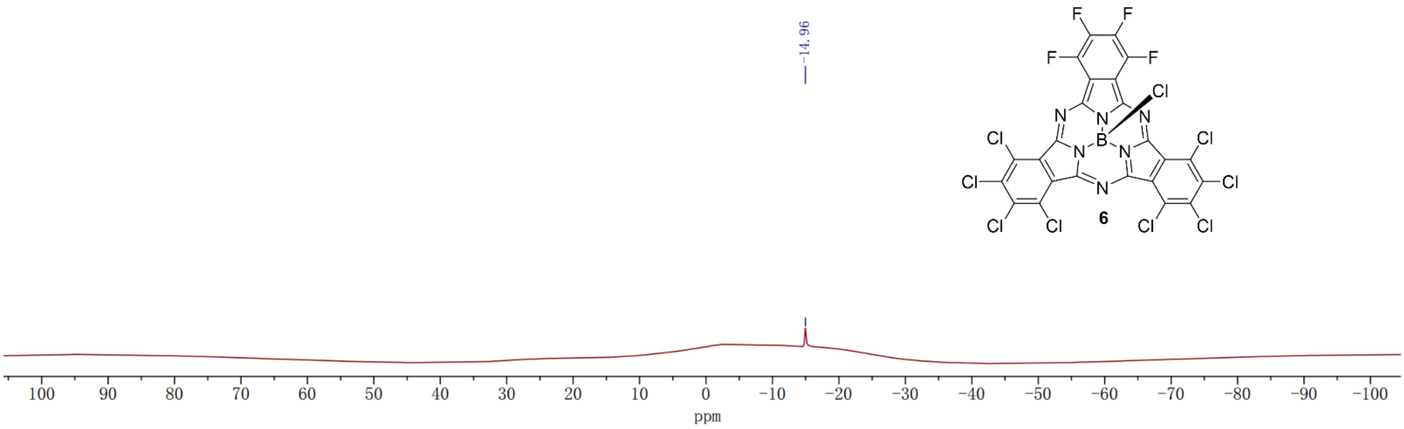

Figure S12. ^{11}B NMR spectrum of **6** in $CDCl_3$.

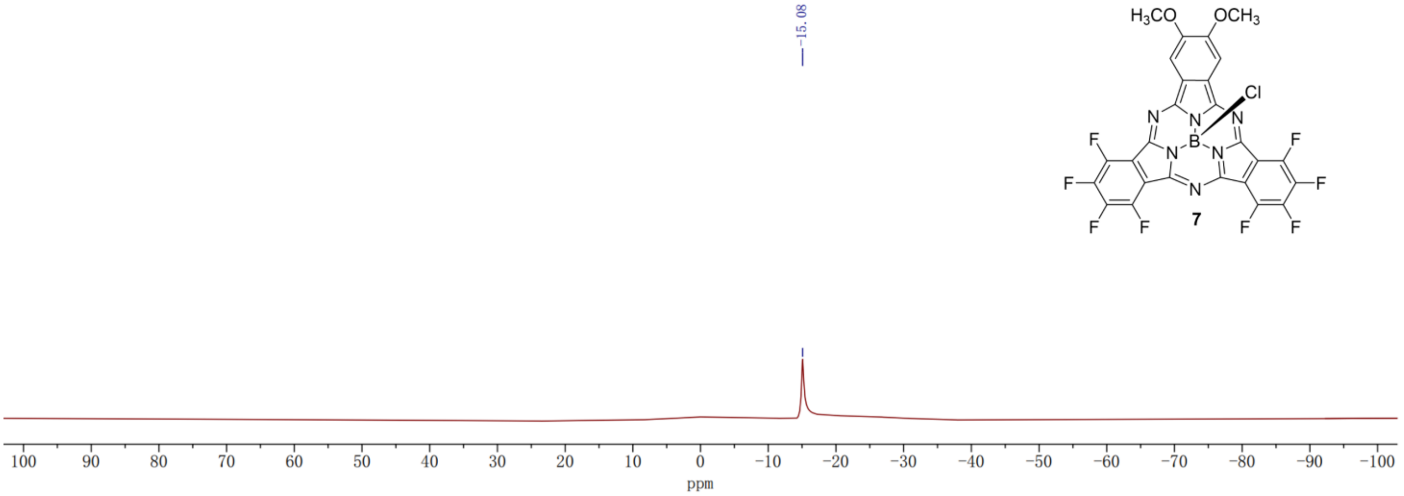

Figure S13. ^{11}B NMR spectrum of **7** in $CDCl_3$ (* residual solvent).

iv. IR Spectra

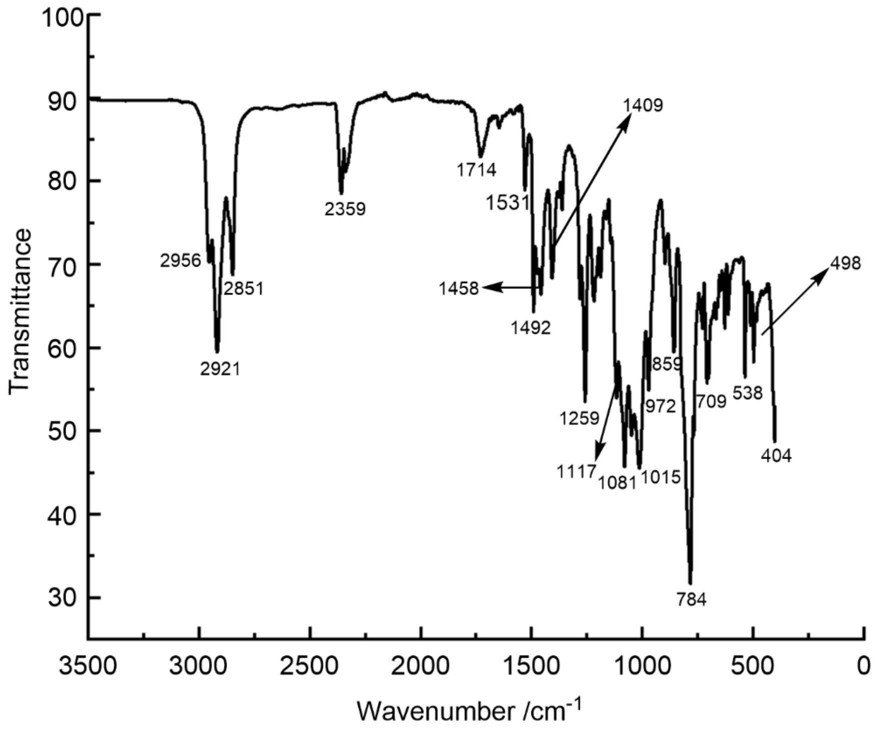

Figure S14. IR spectra of **5** in CH_2Cl_2.

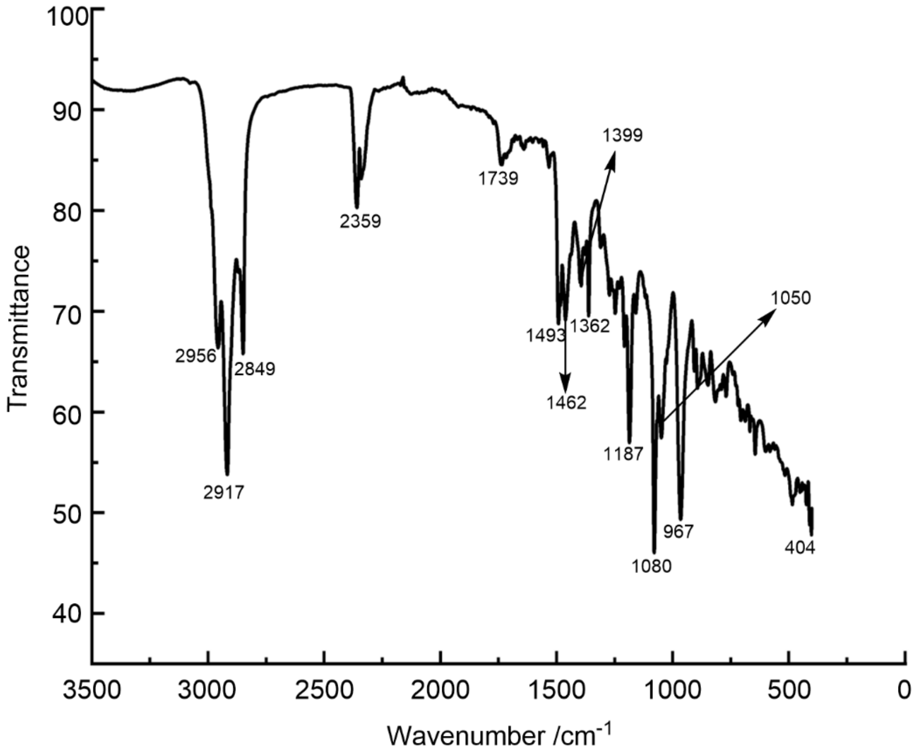

Figure S15. IR spectra of **6** in CH_2Cl_2.

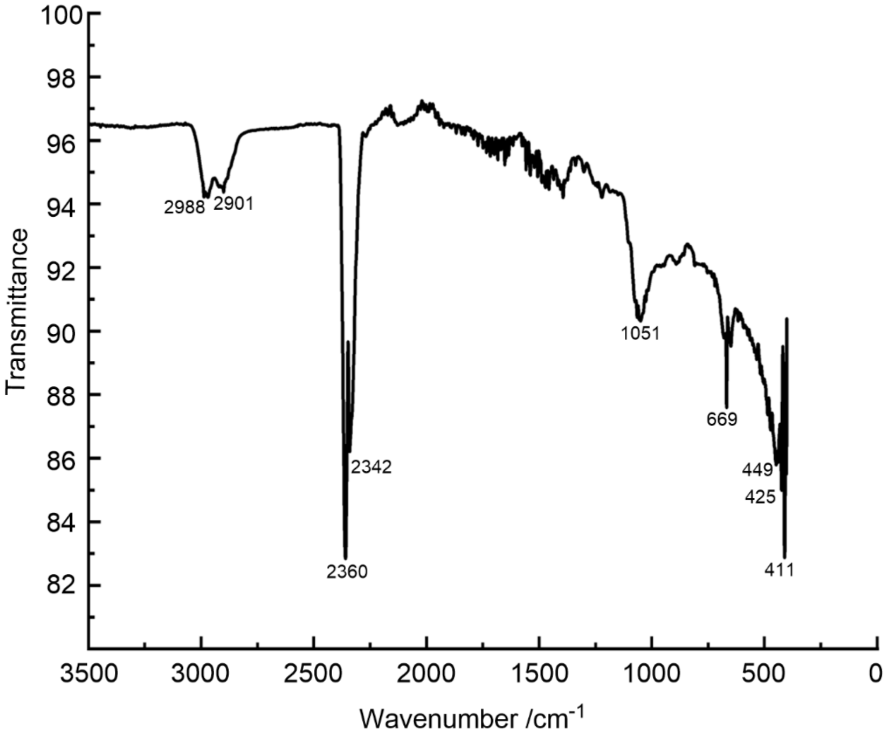

Figure S16. IR spectra of **7** in CH_2Cl_2.

v. Crystallographic data

Table S1. Crystal data and structure refinement.

Moiety formula	$C_{24}BCl_9F_4N_6$	F (000)	804
Formula weight	1643.26	θ (°) for data collection	2.611 to 27.450
Temperature (K)	300	Index ranges	$-9<=h<=9$, $-17<=k<=16$, $-20<=l<=20$
Wavelength (Å)	0.71073	Reflection collected	54438
Crystal system	Triclinic	Unique reflections	6922 (R_{int} = 0.0922)
Space group	P-1	Completeness	0.999
Unit cell dimensions	a = 7.296(2) Å, α = 102.695(9)	Absorption correction	multi-scan
	b = 13.497(4) Å, β = 95.706(11)	Max. and min. transmission	0.7456, 0.6600
	c = 16.141(5) Å, γ = 98.457(9)	Data/restrains/parameters	6922 / 86 / 452
Volume (Å^3)	1519.3(8)	Goodness-of-fit on F^2	1.013
Z	1	Final R indices [$I > 2\sigma(I)$]	0.0534
Density (calcd.) (g/cm^3)	1.796	wR_2 indices (all data)	0.1205
mu (cm^{-1})	0.973	Large diff. peak and hole	0.430, −0.326
Crystal size (mm^3)	0.16 x 0.15 x 0.13	CCDC no.	2220751

Table S2. The length (Å) of C-F bonds and C-Cl bonds from the monocrystal of **6**.

**C1−F1**	**C2−F2**	**C3−F3**	**C4−F4**	**C5−Cl1**	**C6−Cl2**
1.343	1.338	1.351	1.347	1.709	1.726
**C7−Cl3**	**C8−Cl4**	**C9−Cl5**	**C10−Cl6**	**C11−Cl7**	**C12−Cl8**
1.718	1.717	1.709	1.719	1.716	1.711

vi . TD-DFT calculation results

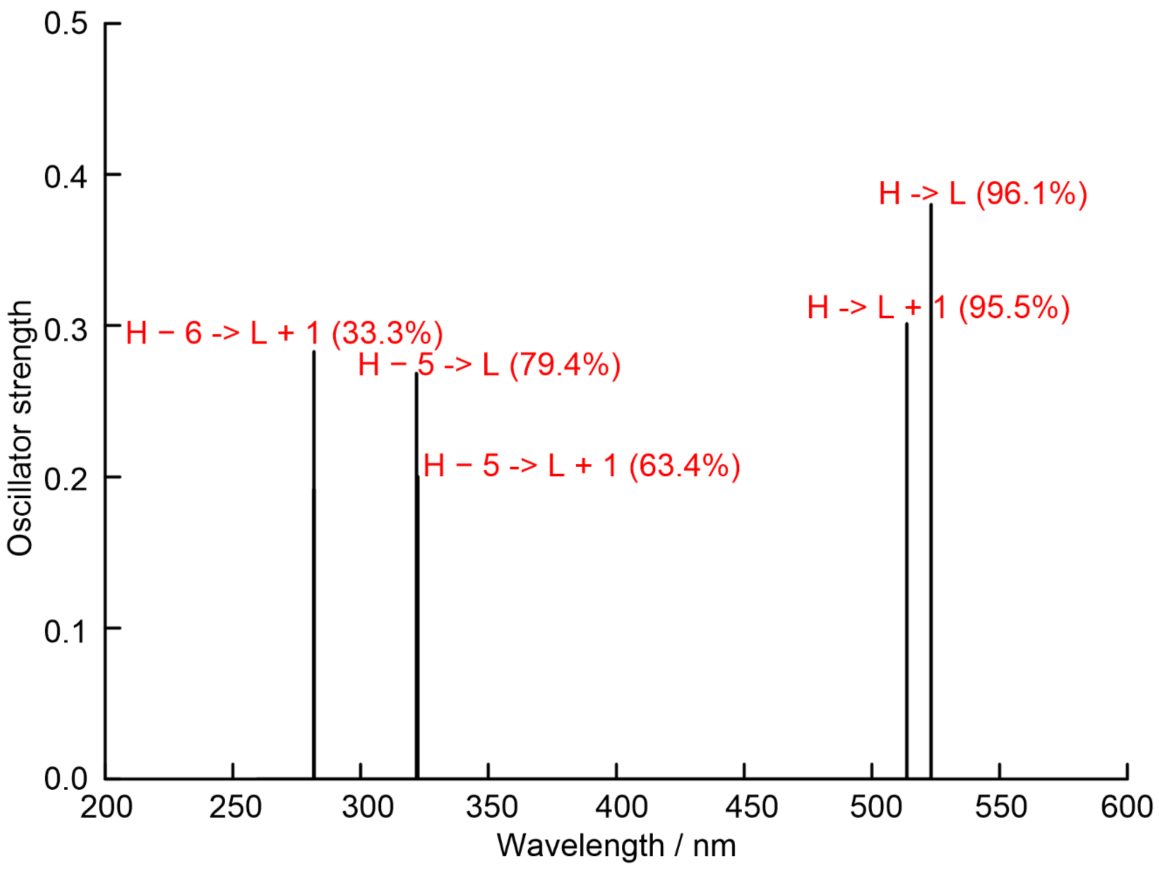

Figure S17. Vertical excitation energy of compound **5** calculated based on TDDFT.

Table S3. Selected transition energies, oscillator strengths, and major contributors of **5** calculated by TDDFT methods at the B3LYP/6-31G(d) level.

**λ(nm)**	***f*[a]**	**Major contribution (weight%)[b]**
523.27	0.38010	H -> L (96.1%)
513.60	0.30130	H -> L + 1 (95.5%)
322.36	0.20020	H − 5 -> L + 1 (63.4%), H − 4 -> L (14.8%), H − 3 -> L+1 (10.1%), H − 6 -> L + 1 (6.8%)
321.74	0.26820	H − 5 -> L (79.4%), H − 6 -> L (6.4%), H − 4 -> L + 1 (6.1%)
281.71	0.28207	H − 6 -> L + 1 (33.3%), H − 7 -> L (25.4%), H − 7 -> L + 1 (9.1%), H − 6 -> L (7.7%)

[a] : Oscillator strength. [b]: H and L denote the HOMO and LUMO, respectively.

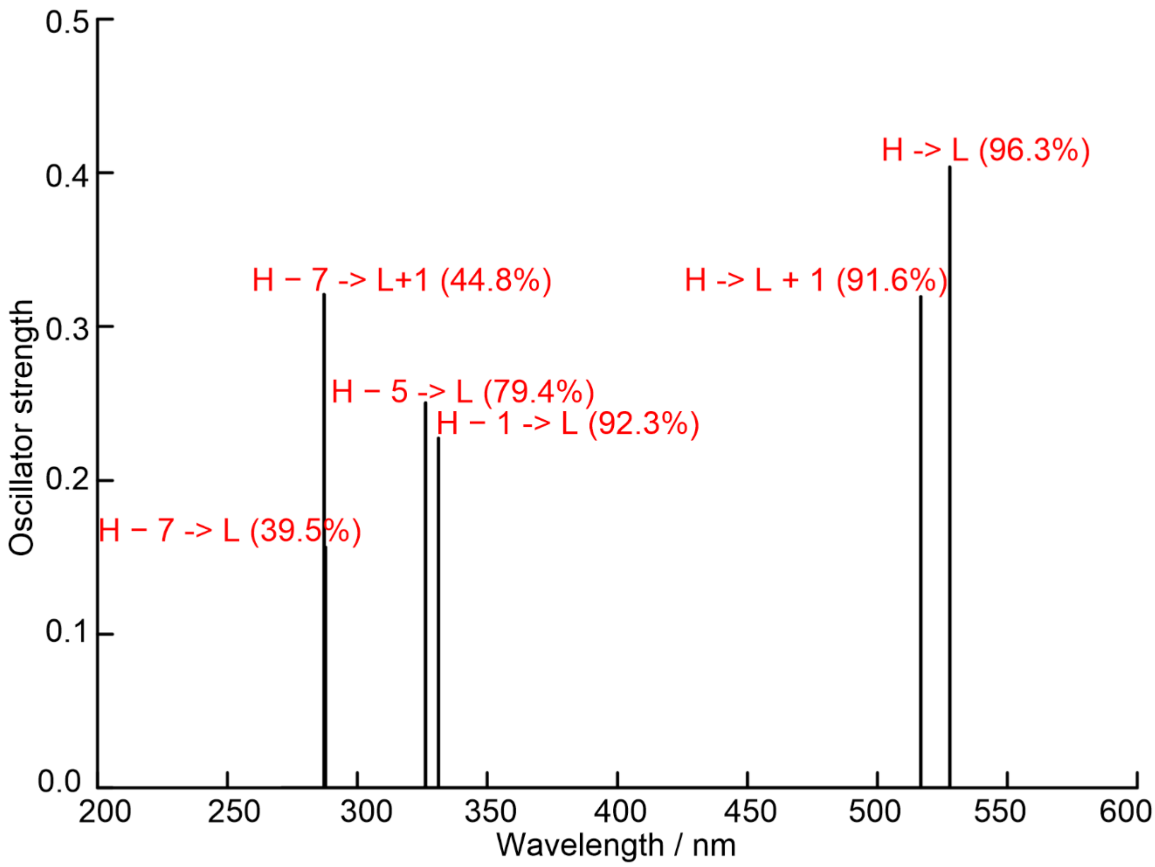

Figure S18. Vertical excitation energy of compound **6** calculated based on TDDFT.

Table S4. Selected transition energies, oscillator strengths, and major contributors of **6** calculated by TDDFT methods at the B3LYP/6-31G(d) level.

λ(nm)	*f*[a]	Major contribution (weight%)[b]
528	0.40380	H -> L (96.3%)
517	0.31960	H -> L + 1 (91.6%)
331	0.22720	H − 1 -> L (92.3%)
326	0.25050	H − 5 -> L (79.4%), H − 3 -> L + 1 (6.8%)
288	0.15660	H − 7 -> L (39.5%), H − 6 -> L + 1 (32.3%), H − 11 -> L (8.6%)
287	0.32090	H − 7 -> L+1 (44.8%), H − 6 -> L (19.6%), H − 8 -> L + 1 (7.2%), H − 11 -> L + 1 (5.9%), H − 5 -> L + 1 (5.1%)

[a]: Oscillator strength. [b]: H and L denote the HOMO and LUMO, respectively.

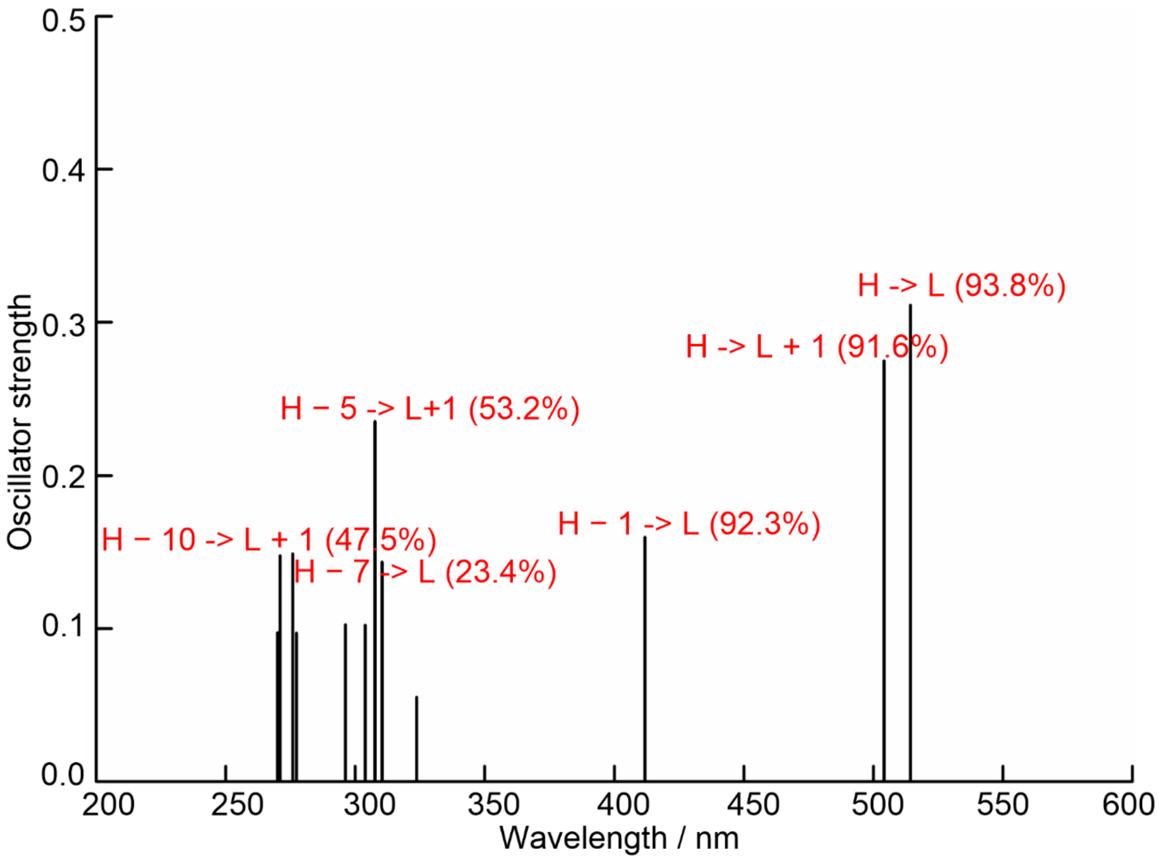

Figure S19. Vertical excitation energy of compound **7** calculated based on TDDFT.

Table S5. Selected transition energies, oscillator strengths, and major contributors of **7** calculated by TDDFT methods at the B3LYP/6-31G(d) level.

λ(nm)	*f*[a]	Major contribution (weight%)[b]
514	0.31140	H -> L (93.8%)
504	0.27510	H -> L + 1 (91.6%)
411	0.15980	H − 1 -> L (92.3%)
308	0.23540	H − 5 -> L+1 (53.2%), H -> L + 4 (15.4%), H -> L + 3 (7.9%), H -> L + 2 (7.7%), H − 4 -> L (5.2%)
276	0.14870	H − 7 -> L (23.4%), H − 6 -> L + 1 (16.7%), H − 7 -> L + 1 (15.3%), H − 8 -> L + 1 (11.1%), H − 10 -> L (9.3%)
271	0.14770	H − 10 -> L + 1 (47.5%), H − 10 -> L (12.1%), H − 6 -> L + 1 (8.2%), H − 1 -> L + 4 (6.0%), H − 1 -> L + 2 (5.2%)

[a] : Oscillator strength. [b] : H and L denote the HOMO and LUMO, respectively.

vii. References

1. Sheldrick, G.M. Experimental phasing with SHELXC/D/E: combining chain tracing with density modification. Acta Crystallographica Section D: Biological Crystallography. **2010**, *66*, 479-85.

2. Yoshioka, S.; Inokuma, Y.; Hoshino, M.; Sato, T.; Fujita, M. Absolute structure determination of compounds with axial and planar chirality using the crystalline sponge method. Chemical science. **2015**, *6*, 3765-8.

3. Sheldrick, G.M. Crystal structure refinement with SHELXL. Acta Crystallographica Section C: Structural Chemistry. **2015**, *71*, 3–8.
